# The indirect effects of cytomegalovirus infection—mechanisms and consequences

**DOI:** 10.1098/rstb.2024.0398

**Published:** 2025-11-06

**Authors:** Tom A. Yates, Helen Payne, Blair L. Strang

**Affiliations:** ^1^ Institute of Health Informatics, University College London, London NW1 2DA, UK; ^2^ Paediatric Infectious Disease, Imperial College London, London SW7 2AZ, UK; ^3^ Division of Immunology, Stellenbosch University, Stellenbosch 7599, South Africa; ^4^ City St George's University of London, London SW17 0RE, UK

**Keywords:** cytomegalovirus, cancer, HIV, tuberculosis, depression, ageing

## Abstract

In this introduction, we summarize the research papers, review articles, opinion pieces and important aspects of the facilitated discussion from the meeting ‘The indirect effects of cytomegalovirus infection: mechanisms and consequences*’* held at the Royal Society, London, on 14–15 October 2024. The term ‘indirect effects’ describes a statistical excess of pathologies seen in people with human cytomegalovirus (CMV) in the absence of histopathological hallmarks of direct CMV tissue damage. This meeting brought together laboratory scientists, paediatric and adult clinical academics, epidemiologists, and trialists, to discuss the latest research on indirect effects, from biological mechanisms to potential clinical consequences. Important questions regarding the impact of CMV remain unanswered in areas important to human health, such as preterm birth and fetal growth restriction, asymptomatic congenital infection, susceptibility to non-CMV infections, cardiovascular and respiratory disease, transplant, cancer and mental health. Further research is needed to better describe the biology and, critically, to robustly quantify its clinical impact and develop interventions to mitigate any harms.

This article is part of the discussion meeting issue ‘The indirect effects of cytomegalovirus infection: mechanisms and consequences*’*.

## 1. Introduction

It is likely that human cytomegalovirus (CMV) has evolved with its host over millions of years [1]. Isolation and characterization of the virus we now know as CMV was led by Margaret Smith and her colleagues in the 1950s [2]. Several strains have now been isolated and studied. However, the diversity of CMV clinical isolates and complexity of the CMV genome is only now being described [3].

CMV is common, with near universal seropositivity in low-income settings and approximately half of adults infected in high-income settings [4,5]. Historically, academic and clinical study of CMV has focused upon congenital infection, transplant and HIV-coinfection. In these populations, CMV end organ disease, i.e. disease involving tissue damage with characteristic histopathological appearances, causes substantial morbidity and mortality, plus significant social and economic costs.

However, by 1989, it had been suggested that, in addition to these direct effects of CMV infection, the presence of CMV was also associated with a range of pathology absent evidence of end organ disease [6]. These so-called ‘indirect effects’ have been claimed to result in an excess of cardiovascular disease, increased susceptibility to co-infections, immunosenescence in the elderly, altered vaccine responsiveness and a bewildering range of other pathologies. Molecular, cellular and immunological mechanisms that might underpin some of these pathologies are increasingly well described. It seems possible that individuals with CMV end organ disease might concurrently suffer from indirect effects. However, the evidence that CMV is causally associated with many of these outcomes remains sparse.

This scientific meeting on the indirect effects of CMV brought together 355 people, who signed up to attend online, and 130 people, who attended in person. Participants included clinicians, industry and academia; in biomedical and translational science; spanning paediatric, adult and elderly medicine; and from both low- and high-income settings. Four themed sessions over 2 days included presentations and debate on child health, immunocompromised adults, susceptibility to tuberculosis, and immunocompetent adults An overview of these sessions and participants is provided as supplementary material [7]. Here we summarize the manuscripts presented in this linked special edition of Philosophical Transactions and major themes arising in discussions at the meeting.

## 2. Work presented in this special edition of Philosophical Transactions

A range of original research, review articles and opinion pieces were commissioned to reflect the topics of the meeting and interests of the participants, from pregnancy and infancy to immunosenescence.

Paarwater *et al.* hypothesize that the indirect effects of CMV on the placenta might impact child health outcomes, and discuss possible immunological mechanisms by which CMV might be transmitted [8].

Schleiss reviews CMV in the context of congenital infection, with a focus on prognostic biomarkers that might guide therapeutic intervention in children with asymptomatic congenital CMV [9].

Johnson *et al.* describe associations between CMV seropositivity and tuberculosis disease in a small observational cohort of children exposed to *Mycobacterium tuberculosis* in the United Kingdom [10]. Stockdale *et al.* investigate the association between CMV and TB disease in two different African populations [11]. Apparently discordant results in these studies may reflect the small sample sizes.

Ellis *et al.* present the protocol of the NIRVANA study, a double-blind, placebo-controlled, phase 2b clinical trial, set in Uganda and South Africa, evaluating whether it is safe to give valganciclovir to adults with CMV viraemia and advanced HIV disease [12]. There will be a pharmacokinetic sub study. The intention is that NIRVANA informs a subsequent RCT of the same intervention, powered for survival.

Labeb *et al.* use flow cytometry and single-cell RNA sequencing to understand how CMV infection shapes immune responses in samples from a cohort of cardiac surgery patients [13].

Doorly *et al.* attempt to estimate causal associations between CMV infection and both cardiovascular disease and all-cause mortality in UK Biobank data [14]. While the authors thought carefully about covariate selection, the analysis does not account for selection effects that might be expected to bias CMV—disease associations towards the null [15,16].

Jackson *et al.* review the humoral response to CMV infection, and how it may relate to ageing and immunosenescence [17]. They touch on the challenges in addressing these questions in both laboratory and clinical studies.

Nicoli *et al.* review how CMV might influence vaccine responses, highlighting, among other topics, reduced immunogenicity in older individuals [18].

Savitz critiques the evidence linking CMV, inflammation and depression, highlighting the challenges in attributing causality and the possibility that these relationships might be bidirectional [19]. Inflammation can clearly impact mood, as evidenced by interferon-induced depression in people receiving treatment for Hepatitis C [20].

Bremke *et al.* focus on the use of checkpoint inhibitors in the treatment of melanoma [21]. They review evidence suggesting CMV positive individuals may have more favourable treatment responses. The authors highlight the importance of distinguishing virus-specific immune responses, tailored to the pathogen, from the virus-driven, cancer-specific response, which can vary with cell type and stroma.

Finally, Naucler *et al.* review contested evidence regards links between CMV and disease progression in people with glioblastoma [22].

## 3. Major themes from discussion at the meeting

### (a) Interventions

Several novel interventions against CMV have emerged recently with others in development. These include antivirals targeting CMV with favourable safety profiles [23] and new CMV vaccines now entering phase three trials [24,25]. We have recently learnt that antenatal CMV screening to identify seroconversion in early pregnancy allows for preventative treatment with valaciclovir and a 70% reduction in transmission of CMV to the fetus [26]. This is an exciting development, but is operationally difficult to deliver and can only prevent congenital CMV attributable to primary CMV infection. Globally, three quarters of congenital CMV is a result of CMV reactivation [27].

It is critical to better understand the indirect effects of CMV infection to know how best to deploy these new tools.

Importantly, new anti-CMV drugs, such as letermovir and marabivir, lack activity against other herpes viruses, which will make it easier to interpret the results of intervention studies. However, narrower antiviral activity may be disadvantageous in immunocompromised people, where additional protection against herpes simplex and varicella zoster viruses may be wanted.

Vaccines are likely to be initially offered to women of childbearing age and transplant recipients, with the intention being to prevent congenital CMV and the morbidity and mortality associated with acquiring CMV during transplantation. In both contexts, CMV end organ damage is well described, although it has become much less frequent in transplant recipients since routine use of pre-emptive or prophylactic CMV active antivirals [28–30].

Clearly, these same populations may also suffer indirect effects. It seems likely that, in the era of near universal CMV prophylaxis, indirect effects make a major contribution to the excess mortality associated with acquiring CMV at solid organ transplant [6,31,32]. The indirect effects of CMV on the fetus are, as yet, unquantified but have potential to include adverse outcomes, such as preterm birth and fetal growth restriction [8].

Decisions about whether to deploy new interventions against CMV more widely will depend on how they are priced, whether herd immunity is needed to prevent congenital CMV, and quantification of the burden of disease caused by the indirect effects of CMV in the general population.

It is important to consider the possibility that CMV infection may offer some benefits, e.g. in promoting maturation of the peripheral immune system and cross protective immunity. The potential benefits of CMV infection were outlined by Prof. Paul Moss in his keynote address and in a thoughtful prior publication [33].

Ultimately, decisions about how these interventions are deployed should be informed by randomized controlled trials [34]. However, as outlined below, basic science and observational epidemiology are needed to inform trial design.

### (b) Heterogeneity

CMV infection exerts profound effects on the immune system, with a high proportion of T cells directed at CMV epitopes [35] and CMV seropositivity a dominant determinant of variability in immune parameters [36]. However, CMV activity varies across the life course, with high levels of viral replication seen during primary infection, reactivations in association with inflammation or immunosuppression [37], and loss of viral control observed with advancing age [38]. Proposed mechanisms for many CMV indirect effects involve viral protein expression [39,40]. Therefore, it seems likely that the extent of lytic replication and/or latent carriage predicts the burden of indirect effects. This may explain why CMV seropositivity appears to be more associated with reduced immunogenicity following vaccination in older adults than in younger vaccine recipients [18]. Those studying the indirect effects of CMV should be mindful of this potential heterogeneity and exercise caution averaging effect estimates across diverse populations.

### (c) Inferring causality

While the biology that may underpin CMV indirect effects is increasingly well described, the real-world consequences remain poorly understood.

There are data from randomized controlled trials in solid organ transplant recipients to support the suggestion that suppressing CMV replication reduces susceptibility to non-CMV infections [30]. Three small randomized controlled trials exploring whether CMV suppression improves outcomes in critically unwell adults reached discordant conclusions [41,42]

Two RCTs in immunosuppressed populations suggest that CMV suppression reverses immunological changes associated with CMV infection [43,44], although one of these studies made analytical errors [45]. The ELICIT trial, reported at the 2025 Conference on Retroviruses and Opportunistic Infections, demonstrated that, in people living with HIV and in receipt of antiretroviral therapy, CMV suppression with letermovir unexpectedly increased levels of soluble tumour necrosis factor receptor 2 (sTNFR2; see [46]). The clinical significance of this finding is unclear, but the result advances our understanding of CMV biology.

Non directed solid organ transplant offers a natural experiment allowing for cautious causal inference. Here, the timing of infection is clear and the only factors relating donor CMV status—the overwhelming determinant of acquiring CMV at transplant—and recipient characteristics are the organ allocation algorithm and calendar time. As such, confounding is tractable. Analyses of large cohorts of US kidney and liver transplant recipients suggest that, even in the era of routine CMV prophylaxis, acquiring CMV at transplant is associated with excess mortality [31,32]. A number of analyses have suggested potential mechanisms for the excess mortality. However, none of these analyses properly account for competing events. It may be reasonable to generalize findings in transplant recipients to primary infection in similarly immunocompromised populations. Furthermore, analyses in transplant recipients arguably provide an upper bound on the extent of indirect effects we might expect in the general population.

In observational studies, extending inference linking CMV indirect effects to disease in the general population is more challenging.

Where CMV serostatus is used as a marker of exposure to CMV, analyses must consider that individuals with CMV are likely to differ systematically from individuals who do not have CMV. For example, in high income countries, individuals with CMV are more often poor, members of ethnic minorities, or born overseas [47–49]. In low-income countries, where CMV is ubiquitous, the small minority that are CMV seronegative after the first year of life may be atypical. In both settings, baseline immunity or social contact patterns may differ between CMV seropositive and CMV seronegative individuals. These variables are challenging to measure and, therefore, challenging to adjust for in statistical models. In observational analyses, mismeasurement of key confounding variables can meaningfully alter conclusions [50]. Importantly, it is usually not possible to know whether conditional exchangeability has been achieved.

Measures of CMV activity, such as DNAemia, are often used as proxies for CMV activity. Here, in addition to addressing confounding, we need to determine directionality, as immunosuppression or incipient disease can result in CMV replication. For example, it remains unclear, in the context of advanced HIV disease, whether the association between CMV DNAemia and mortality is causal [51]. An alternative explanation for this observation is that CMV DNAemia is a marker of immunosuppression. This ‘chicken versus egg’ question came up repeatedly in discussion at the meeting.

If we were only interested in large effects, it would matter less that we could not confidently exclude small degrees of residual bias. However, because CMV infection is ubiquitous in much of the world, small perturbations in susceptibility to cardiovascular disease or non-CMV infections could have a large population impact.

There are study designs that might help address some of these issues.

Case only methods perfectly account for fixed between individual differences [52]. However, they require sufficiently granular longitudinal measurement of exposure and only work if there is variability in the exposure during the period of life when the outcome occurs. Moreover, the method only accounts for fixed differences, and analysts may still not be sure they have adequately accounted for confounding by variables, such as social contact patterns, that can change over time.

Mendelian randomization should address both potential reverse causation and classical confounding [53]. However, the assumptions are violated where genetic variants that predict the exposure might independently impact the outcome, so-called ‘horizontal pleiotropy’. This seems likely when seeking to understand whether CMV infection predisposes to non-CMV infections. Furthermore, while associations between genetic variants and CMV seropositivity have been reported [54], as yet there is no genome wide association study where the ability to control CMV is the outcome.

Vaccine probe studies are another potentially robust approach, and have been used successfully to understand the contribution that other pathogens make to disease burden [55]. However, where receipt of vaccine is not randomized, careful adjustment for variables that predict both receipt of vaccine and the outcome of interest is required. Furthermore, despite some previous partially successful vaccine trials, and a number of new vaccines now entering Phase 3 trials, there are no proven CMV vaccines currently available [24,25].

Given the challenges in demonstrating causality in observational analyses, it is encouraging to see randomized controlled trials emerging to provide definitive answers regards CMV indirect effects. Three trials with clinical endpoints are ongoing. In all, the intervention is ganciclovir or its oral prodrug valganciclovir. GRAIL^3 (NCT04706507) will test whether suppressing CMV replication reduces duration of intubation in adults with severe pneumonia. VIGAS2 (NCT04116411) asks whether suppressing CMV replication improves overall survival in people with glioblastoma. The phase 3 trial planned by the NIRVANA investigators will ask whether suppressing CMV replication improves survival in adults with advanced HIV disease [12].

Given small reductions in the indirect effects of CMV could have a large impact on population health, future intervention studies should be powered to detect modest effect sizes. As such, these trials will need to be large [56]. In designing randomized controlled trials, and choosing outcomes, investigators should prioritize interventions with biological plausibility, where observational studies using methods with different potential biases suggest benefit is likely [57].

### (d) Cross disciplinary collaboration

CMV’s indirect effects manifest across multiple organ systems and disease pathways, requiring insights that go beyond any single scientific discipline. A striking feature of discussions at this meeting was that many researchers were unaware of relevant work occurring outside their immediate areas of study. For instance, scientists investigating CMV-driven immune modulation in solid organ transplant recipients were not aware of research in African children suggesting that CMV infection may increase susceptibility to tuberculosis [58]. Similarly, reproductive immunologists studying maternal–fetal transmission had limited contact with investigators analysing cardiovascular or neurocognitive sequelae of CMV in adults. This fragmentation underscores the need for structured, cross-disciplinary networks where ideas, datasets and methodologies can be shared.

## 4. Summary and future directions

This meeting and the works contained within this special edition demonstrate the complex interactions between CMV and its human host. Given the virus is so prevalent, modest indirect effects could have a considerable impact on population health. With interventions against CMV increasingly available, there is an urgent need to better characterize this biology, to quantify causal associations between CMV and specific health outcomes, and to develop and robustly evaluate interventions to mitigate any harms.

Regular multidisciplinary meetings, joint training schemes and open-access data repositories could help bridge disciplinary divides and accelerate hypothesis testing across clinical and laboratory domains. Investment in cross-disciplinary infrastructure such as shared biobanks, clinical cohorts and protocols, would also strengthen our collective ability to identify, quantify and ultimately mitigate the indirect effects of CMV.

Future collaborations should prioritize equity and capacity-building in low- and middle-income countries, where the burden of CMV-associated disease is greatest. Empowering researchers in resource-limited environments can ensure that questions of global relevance are addressed, using locally meaningful data. Supporting early-career researchers, especially those in under-represented regions, will be essential to sustain momentum in research into the indirect effects of CMV.

## Data Availability

This article has no additional data. Supplementary material is available online [7].
